# Modern Brain Mapping – What Do We Map Nowadays?

**DOI:** 10.3389/fpsyt.2015.00089

**Published:** 2015-06-16

**Authors:** Maria Nazarova, Evgeny Blagovechtchenski

**Affiliations:** ^1^Centre for Cognition and Decision Making, National Research University Higher School of Economics, Moscow, Russia; ^2^Research Center of Neurology, Moscow, Russia; ^3^Institute of Translational Biomedicine, St. Petersburg State University, St. Petersburg, Russia

**Keywords:** brain mapping, motor control, sensory systems, history of brain mapping, function localization

## Introduction

The problem of function localization in the brain is one of the most fundamental in neuroscience. There are two opposite paradigms relating to the problem: “modularism,” also known as “localism,” versus “holism,” which have been discussed for a long time (1, 2). The debate in favor of one or another view can still be traced at all methodological levels – from the cell to the system. In this opinion paper we want to raise a question – what is meant nowadays by brain mapping? In addition, we want to highlight the necessity of being aware of occasionally occurring discontinuity in the research at different methodological scales. This problem is evident for experts in the field, but not always sufficiently so for early career researches. We will try to describe the difficulties of modern brain mapping primarily by looking at one of the currently best-studied functions – motor function.

## History of Opposition of Modular Versus Holistic Conceptions of Brain Organization

A somewhat artificial opposition of “modular” and “holistic” organization of the brain has been evident in neuroscience from 18th century, and started mostly as a disagreement between physiologists working on animals and clinicians studying brain lesions in humans (1–3). A first revision of the term “function” by a clinician and a step away from hardwired localism was performed by the neurologists J. H. Jackson at the end of 19th century who wrote that “localization of a symptom is not localization of a function” (4). In the beginning of the 20th century, a paradigm shift occurred toward gestalt psychology, which changed the trend of research at the macro-scale level towards a more holistic view (2). A prime example of a confrontation at the micro-scale at the same time was the debate of Golgi and Cajal regarding the essence of a neuron (5). In 1937, a neurosurgeon W. Penfield performed the first cortical cartography in humans and published an iconic description of sensory and motor homunculi (6). In the second quarter of the 20th century, the concept of a function as a goal-dependent entity appeared in the form of theory of movements (7) and theory of functional systems (8), both viewing a function as a non-rigid goal-dependent entity. To date, it is usually postulated that localism and holism have been replaced by “connectionism,” with many studies nowadays trying to find interactions between brain regions and not the function of these regions by themselves (9, 10). However, it seems there is still a tendency to favor localism, especially in the cognitive sciences (11, 12). Perhaps, this is due to the fact that modern non-invasive methods, such as PET, fMRI or TMS, are mostly associated with functional mapping of the brain based on M. Minsky’s philosophy that “minds are what brains do.”

## Discontinuity of the Motor Research of Different Methodological Scales

The question remains open of how cortical “activation” at the macro-level, viewed for example with fMRI or EEG, is linked to micro-scale phenomena such as single neuron activity in the spinal cord in awake animals (especially in humans) (13, 14). There is a large community of researches studying ways of activation of a particular alpha-motor neurons in the spinal cord (15–17); scientists working on the level of a single neuron usually associate it to a specific task (16, 18). Thereby, those who work with slices of the spinal cord are well aware of how to activate a certain motoneuron (19), but it is still difficult to bridge these phenomena with activation of the cortex (15). Modern macro-scale approaches connecting peripheral and central recording, such as TMS-EEG and corticomuscular coherence, including biofeedback, are trying to overcome this gap (20).

A good example of a discontinuity in motor research at different methodological scales is the phenomena of convergence and divergence of motor cortex organization. They are well known in micro- and meso-scale studies. For example, in the invasive brain–computer interface (BCI) research, principles like neural degeneracy and neuronal multitasking were formulated (21). However, these phenomena are still widely overlooked in the research at the macro-scale level (22). For instance, a commonly used term in macro-scale research is an “area of a muscle cortical representation” (23, 24), which is suitable for practical use like presurgical motor mapping (25), but is physiologically dubious considering the proven fact that some pyramidal cells may broadly innervate corresponding alpha motoneurons relating to activation of different muscles, even of different limb segments (26–28).

## Goal-Driven Concept of a Function – Problem of Awake Versus Anesthetized Animals

Clearly demonstrated in many studies, context and goal-dependency of a motor function (29, 30) brings us back to the necessity to revise the concept of a function as an environment and goal-dependent entity. Since a large amount of classical mapping experiments were performed on anesthetized animals, where conditions are stable and there is no context or goal directed behavior, one should be careful when interpreting the results of these experiments. The data obtained in anesthetized animals are widely used as a teaching material: the construction of the motor and sensory homunculus is an essential part of most neuroscience textbooks (31, 32). However, the experiments on awake animals raise doubts about this rigid structure (33).

## Renaissance of a Goal-Driven Concept of a Function in Neurorehabilitation

The problem of function localization in the brain is relevant for the development of new approaches to rehabilitation after brain damage. Hence, in the 80s and 90s, the popularity of the idea of long-term brain reorganization resulted in the birth of neurorehabilitation as a new and fruitful field (34, 35). Nowadays, the growing understanding of the on-line instability and goal-dependency of a function is bringing new trends to this field. Thus, new approaches in neurorehabilitation are oriented not toward simple movement training but toward the recovery of a whole goal-oriented action (36, 37). The renaissance of a systematic view on function resulted in the appearance of new techniques, comprised of simultaneous application of many modalities, including visual biofeedback such as mirror therapy (38, 39) or multimodal biofeedback during motor rehabilitation (40).

Understanding function in a corresponding context necessitates the development of closed-loop approaches of therapeutic brain stimulation (like TMS), instead of stimulation of different cortical regions at rest. These closed-loop approaches require the elaboration of protocols for central-peripheral stimulation coupling for the optimal modulation of the recovering brain (41, 42). Such task-dependent closed-loop approaches can be combined with brain-state guided stimulation. The last few years have witnessed an increase in studies identifying brain-states favorable for stimulation, for example based on pre-stimulus EEG (43, 44). This is going to lead to the development of new protocols for therapeutic stimulation with better timing. A similar methodology is already successfully used in the field of invasive BCI combining brain activity recording with cortical stimulation (45, 46). Favorable preconditioning brain-states could also be deliberately achieved using approaches such as transcranial alternating current stimulation (tACS), allowing brain stimulation with a specific frequency during a task (47). The dynamic nature of a function makes evident the impossibility of a one-size-fits-all-strategy (48), and highlights the need for dynamic revision of the targets and strategies throughout the recovery process in brain damage patients.

## Conclusion

Despite the fact that the field is moving from the holism–localism opposition toward a paradigm of connectionism, which can be seen in the development of such big projects as Connectome or Human Brain Project (49), we still lack a clear understanding of how to address the problem of functions localization. We wanted to highlight the importance of being careful with extrapolations based on the *a priori* assumptions, which appear sometimes to be different at different methodological scales. A promising new approach which may lead to a new understanding of how to distinguish networks and areas in the brain may be optogenetics, a method allowing the stimulation not of the specific regions of the brain, but of the specific networks and inputs (50). Another encouraging trend is the growing BCI field which combines observational and interventional approaches (21).

## Conflict of Interest Statement

The authors declare that the research was conducted in the absence of any commercial or financial relationships that could be construed as a potential conflict of interest.
